# The Use of ‘Omics for Diagnosing and Predicting Progression of Chronic Kidney Disease: A Scoping Review

**DOI:** 10.3389/fgene.2021.682929

**Published:** 2021-11-08

**Authors:** Melanie A. Govender, Jean-Tristan Brandenburg, June Fabian, Michèle Ramsay

**Affiliations:** ^1^ Division of Human Genetics, National Health Laboratory Service and School of Pathology, Faculty of Health Sciences, University of the Witwatersrand, Johannesburg, South Africa; ^2^ Sydney Brenner Institute for Molecular Bioscience, Faculty of Health Sciences, University of the Witwatersrand, Johannesburg, South Africa; ^3^ Wits Donald Gordon Medical Centre, School of Clinical Medicine, Faculty of Health Sciences, University of the Witwatersrand, Johannesburg, South Africa

**Keywords:** ‘omics, biomarkers, early detection, diagnosis, Sub-Saharan Africa, diabetes, chronic kidney disease, hypertension

## Abstract

Globally, chronic kidney disease (CKD) contributes substantial morbidity and mortality. Recently, various ‘omics platforms have provided insight into the molecular basis of kidney dysfunction. This scoping review is a synthesis of the current literature on the use of different ‘omics platforms to identify biomarkers that could be used to detect early-stage CKD, predict disease progression, and identify pathways leading to CKD. This review includes 123 articles published from January 2007 to May 2021, following a structured selection process. The most common type of ‘omic platform was proteomics, appearing in 55 of the studies and two of these included a metabolomics component. Most studies (*n* = 91) reported on CKD associated with diabetes mellitus. Thirteen studies that provided information on the biomarkers associated with CKD and explored potential pathways involved in CKD are discussed. The biomarkers that are associated with risk or early detection of CKD are SNPs in the *MYH9/APOL1* and *UMOD* genes, the proteomic CKD273 biomarker panel and metabolite pantothenic acid. Pantothenic acid and the CKD273 biomarker panel were also involved in predicting CKD progression. Retinoic acid pathway genes, *UMOD*, and pantothenic acid provided insight into potential pathways leading to CKD. The biomarkers were mainly used to detect CKD and predict progression in high-income, European ancestry populations, highlighting the need for representative ‘omics research in other populations with disparate socio-economic strata, including Africans, since disease etiologies may differ across ethnic groups. To assess the transferability of findings, it is essential to do research in diverse populations.

## Introduction

The global prevalence of chronic kidney disease (CKD) is estimated to be 13.4%, and thought to be higher in low- and middle-income settings, such as sub-Saharan Africa (SSA) (15.8%) (Hill et al., 2016; Kaze et al., 2018). End stage kidney disease (ESKD) is the most severe stage of CKD, at which point the damage is irreversible and support in the form of chronic dialysis or a kidney transplant is required to sustain life. Major risk factors for CKD vary by setting, with hypertension (HT) being the most common in east Asia, tropical Latin America, and Western and Southern SSA, whereas diabetes predominates as a risk factor in the remaining regions of the world (Bikbov et al., 2020). Endemic and other infectious diseases, for example schistosomiasis, malaria, and human immunodeficiency virus (HIV) still contribute substantial additional risk for CKD in high prevalence countries (Couser et al., 2011; Ekrikpo et al., 2018). The diagnosis of CKD is currently based on estimated glomerular filtration rate (eGFR) and/or albuminuria (Levey et al., 2002). However, low GFR defined as eGFR<60 ml/min/1.73 m^2^, is a relatively late marker of kidney disease, as organ damage may precede functional changes such as filtration impairment. Even though albuminuria is suggested to be an early marker of kidney disease, kidney disease may still progress despite the absence of albuminuria (Miller et al., 2009). Thus, there is a need for alternative markers of kidney disease that identify early disease and predict risk for progression. Such markers would enable interventions to prevent or slow progression of CKD, which is particularly relevant in resource-limited settings where access to kidney replacement therapy for ESKD remains severely restricted.

‘Omics technologies refer to collective analyses of cell populations, tissues, organs or the whole organism at the molecular level (Horgan and Kenny, 2011). In the last decade, there have been significant advances in use of ‘omics platforms to investigate susceptibility to, and detection of CKD, and related risk factors (Good et al., 2010; Abbiss et al., 2019; Cañadas-Garre et al., 2019). ‘Omics technologies permit high-throughput, comprehensive exploration of the genome (DNA sequence), epigenome (epigenetic modifications), proteome (proteins), transcriptome (transcribed RNA) and metabolome (metabolites), through a wide range of platforms including next-generation sequencing, protein and mRNA arrays and mass spectrometry (Papadopoulos et al., 2016). The past decade has seen significant advances in genome-wide association studies (GWAS) for CKD and kidney function traits, with study sample sizes increasing from >100,000 to 1,000,000 facilitating the identification of >250 genetic loci robustly associated with CKD in different ethnic groups (Tin and Köttgen, 2020). Proteomic approaches to identify urinary biomarkers for early detection and prediction of progression of CKD are also on the rise, attractive (in part) for their non-invasive use in clinical settings (Mischak et al., 2010). For example, capillary electrophoresis coupled with mass spectrometry (CE-MS) has been used to develop a proteome-based urine biomarker panel of 273 peptides with profiles that differed significantly between individuals with CKD and healthy controls (Good et al., 2010). These peptides were combined to develop a single score known as the CKD273 risk score (Good et al., 2010). Previous studies defined a CKD273 risk score cut-off of 0.343 to predict diabetic nephropathy (DN) (Alkhalaf et al., 2010). This threshold was lowered to 0.154 to accommodate the early detection of CKD (Lindhardt et al., 2016).

The overall aim of this scoping review was to evaluate existing literature for potential biomarkers–aside from those currently used, namely eGFR and albuminuria–that might facilitate early detection of CKD, and predict its progression. The specific objectives were to 1) describe potential biomarkers identified through different ‘omics platforms (genomics, proteomics, metabolomic, transcriptomics and epigenomics) that are associated with CKD, and 2) to explore potential pathways involved in CKD that could inform novel treatment strategies for CKD. This is pertinent for developing affordable diagnostic, prevention, and therapeutic strategies for countries with high prevalence of CKD and limited resources, such as are found in SSA.

## Methods and Materials

### Protocol Registration

This review was conducted using the Preferred Reporting Items for Systematic reviews and Meta-Analyses extension for Scoping Reviews (PRISMA-ScR) approach (de Oliveira, 2018). The background and methods were documented in a protocol in the Open Science Framework (OSF) database (Doi:10.17605/OSF.IO/QM64P, https://osf.io/sw8ne/).

### Search Strategy

Relevant studies published from January 2007 to 18 May 2021 (Search date: 18 May 2021) were identified through a comprehensive search of electronic databases including Embase, Web of Science, PubMed, SCOPUS and African Index Medicus. The electronic database search was conducted using medical subject heading (MeSH) terms, keywords pertinent to the topic, and Boolean operators such as AND/OR to obtain the maximum number of studies (Table 1).

**TABLE 1:** Search strategy used in database searches.

Search no	Search query
#1	“chronic kidney disease” OR “CKD” OR “GFR” OR “glomerular filtration rate” OR “nephropathy” OR “serum creatinine” OR “urinary albumin” OR “ACR” OR “albumin creatinine ratio” OR “albuminuria” OR “urinary albumin creatinine ratio” OR “UACR” OR “proteinuria” OR “protein creatinine ratio” OR “urinary protein creatinine ratio” OR “UPCR”
#2	“diabetes” OR “hypertension” OR “infection” OR “infectious disease” OR “HIV” OR “human immunodeficiency virus” OR “malaria” OR “schistosomiasis” OR “cytomegalovirus” OR “CMV” OR “JC virus” OR “John Cunningham virus” OR “polyoma virus” OR “*tuberculosis*” OR “TB” OR “hepatitis B virus” OR “hepatitis C virus”
#3	“genomic” OR “metabolomic” OR “proteomic” OR “transcriptomic” OR “epigenomic”
#4	1# AND #2 AND #3

### Screening and Eligibility Criteria

Database search records were combined and all duplicate records removed. One investigator (MG) screened all records by title and abstract for inclusion, and articles with irrelevant topics were excluded. Investigators (J-TB and JF) independently screened records by title and abstract for verification. Any inconsistencies or disagreements were resolved by review with investigator MR. This was followed by screening full-text articles according to the eligibility criteria. The main inclusion criterion was that a study had to be an original study containing ‘omics data used to detect CKD and related risk factors. The studies had to report on the characteristics of the study population, type of ‘omics technique used, CKD or kidney function traits, relevant risk factors (defined in Table 1), and potential biomarker or indicator of early detection of CKD and prediction of progression (methylation profile, genes/genetic variants, transcripts, proteins and/or metabolites). Since the first large GWAS was conducted in 2007 (Craddock and Jones, 2007), we chose to include only studies published from January 2007 to May 2021. The age limit was restricted to participants 18 years and older and only studies published in English were included. Studies were excluded if the study was a review, meta-analysis, conference abstract or presentation, book chapter or response to treatment. Studies involving animals and paediatric participants were also excluded. The reasons for excluding each article were recorded.

### Data Extraction

A data extraction sheet was created on Microsoft Excel (version 16.42, available at: https://office.microsoft.com/excel) to ensure all relevant data were extracted. Data extraction was performed by MG, and any discrepancies were resolved by J-TB, JF and MR. Data extracted for each article included the digital object identifier (DOI), name of first author, year of publication and title, participant/population characteristics (sample size, ethnicity, stage of CKD, presence of risk factors and/or infections), type of ‘omics technology, ‘omics technique, biological sample and potential biomarkers (genes, variants, miRNAs, mRNAs, proteins, and peptides) associated with early detection of CKD and prediction of progression. As PRISMA-ScR guidelines recommend, we conducted a qualitative synthesis to summarise the main components of the CKD ‘omics field.

## Results

**FIGURE 1:**
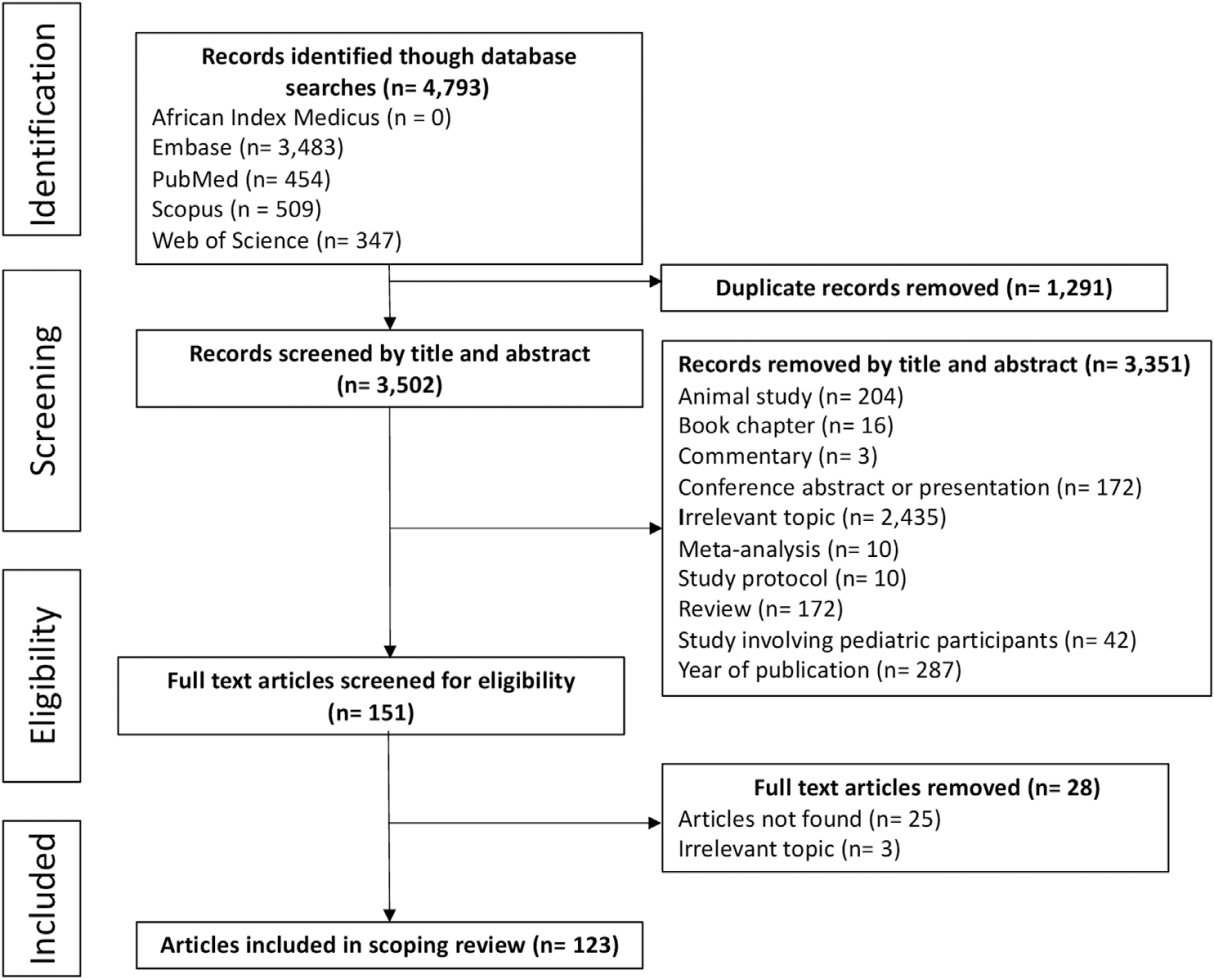
Article selection process according to PRISMA-ScR guidelines.

The database searches identified 4,793 articles, of which 1,291 were duplicates. Through title and abstract screening, we excluded 3,351 articles mainly due to the following reasons: irrelevant topic, year of publication outside the period for this review, and animal studies. A further 28 articles were excluded after assessing the full-text and applying the eligibility criteria. Thus, one hundred and twenty-three full-text articles were included in this review (Figure 1, Supplementary Appendix A).

### Participant and Study Characteristics

The participant and study characteristics of all studies (*n* = 123) included in this scoping review are shown in Table 2. There was an increase in publications from 2015. Majority of studies (28.5%) had sample sizes greater than 500. Most of the participants (42.3%) were between 51 and 60 years of age. Most of the studies (79.7%) included male and female participants. The most common ethnic group represented was European ancestry (15.4%), followed by Asian (12.2%), African ancestry (6.5%), Indian (3.3%), and studies reporting on multiple ethnicities (10.6%). It is important to note that ethnicity was not reported in over half of the studies (52.0%). Of eight studies that included participants of self-reported African ethnicity, only one included African individuals residing in Africa (Chen et al., 2007).

**TABLE 2:** Participant and study characteristics reported in 123 studies.

Characteristic	Number of studies *n* (%)
**Year of publication**	
2007–2010	22 (17.9)
2011–2015	28 (22.8)
2016–May 2021	73 (59.3)
**Number of participants per study**	
1–10	2 (1.6)
11–50	22 (17.9)
51–100	20 (16.3)
101–200	26 (21.1)
201–500	18 (14.6)
>500	35 (28.5)
**Mean age of participants**	
18–30 years	0 (0)
31–40 years	11 (8.9)
41–50 years	11 (8.9)
51–60 years	52 (42.3)
61–70 years	32 (26.1)
>70 years	2 (1.6)
Unknown	15 (12.2)
**Sex**	
Males only	5 (4.0)
Females only	0 (0)
Both	98 (79.7)
Not stated	20 (16.3)
**Participant ethnicity**	
Asian only	15 (12.2)
African ancestry only	8 (6.5)
European ancestry only	19 (15.4)
Indian only	4 (3.3)
Studies including multiple ethnicities (European ancestry, African ancestry, Hispanic, Indian and Asian)	13 (10.6)
Unknown	64 (52.0)

### Omics’ Platforms and Techniques

We reported on five different ‘omics technologies including genomic, epigenomic, proteomic, metabolomic and transcriptomic (Table 3). The proteomic approach was the most common ‘omics platform (*n* = 53), followed by metabolomics (*n* = 30), genomics (*n* = 17), epigenomics (*n* = 10), transcriptomics (*n* = 5) and eight studies used a combination of ‘omics platforms. A wide range of techniques were used to analyse the ‘omics data (Table 3). Genomic analysis was mainly performed by arrays including Illumina’s High-throughput Bead array, Human Core Exome Bead array, HumanHap550-Duo Bead Chip and Omni Express Exome array. Proteomic analysis was mainly done by liquid chromatography coupled with mass spectrometry (LC-MS) and CE-MS. Gas chromatography-mass spectrometry (GC-MS) and LCMS were important analytical techniques for metabolomic analysis. Methylation arrays (including Illumina’s Infinium 27, 450 and 850K methylation arrays) were key for epigenomic analysis and transcriptomic analysis was mainly performed by RNA sequencing.

**TABLE 3:** The different ‘omics technologies and techniques reported in this scoping review for detection of CKD and associated risk in 123 studies.

Type of omic technology	Number of studies *n* (%)
**Genomic**	**17 (13.8)**
Affymetrix 370K and 500k arrays	
Illumina High-throughput Bead array	
Illumina HumanCoreExome Bead array	
Illumina HumanHap550-Duo BeadChip	
Illumina’s IV panel	
llumina OmniExpressExome array	
Panel of 372 autosomal short tandem repeat markers	
Variety of GWAS arrays	
**Epigenomic**	**10 (8.1)**
Illumina Infinium 27K methylation array	
Illumina Infinium HumanMethylation450 Beadchip	
Illumina Infinium MethylationEPIC BeadChip (HM850K)	
Reverse phase high pressure liquid chromatography (RP-HPLC) analysis and bisulfite sequencing	
SuperTAG methylation-specific digital karyotyping	
**Proteomic**	**53 (43.1)**
1 and 2-dimensional gel electrophoresis (1 and 2DE) and mass spectrometry (MS)	
Capillary electrophoresis coupled with mass spectrometry (CE-MS)	
Fluorescence two-dimensional (2-D) differential in-gel electrophoresis (DIGE)	
Isobaric tag for relative and absolute quantitation iTRAQ MS	
Liquid chromatography coupled with mass spectrometry (LC-MS)	
Matrix-assisted laser desorption/ionization mass spectrometry (MALDI-MS)	
Olink cardiovascular panel	
Protein chip arrays	
Proximity Extension Assay (PEA)	
Surface-enhanced laser desorption/ionization mass spectrometry (SELDI-MS)	
**Metabolomic**	**30 (24.4)**
Commercial kits	
CE-MS	
Gas chromatography coupled with mass spectroscopy (GC-MS)	
LC-MS	
Nuclear magnetic resonance (NMR)	
**Transcriptomic**	**5 (4.1)**
GeneChip human genome series U133A and Plus 2.0 Array	
RNA sequencing	
**Genomic and metabolomic**	**2 (1.6)**
Infinium Multi-Ethnic Global BeadChip array	
Exome BeadChip array	
LC-MS	
**Genomic and epigenomic**	**1 (0.8)**
Illumina Human MethylationEPIC BeadChip	
**Proteomic and metabolomic**	**2 (1.6)**
ELISA Assays	
LC-MS	
SOMAscan	
**Transcriptomic and epigenomic**	**1 (0.8)**
Illumina 450K methylation array	
RNA sequencing	
**Transcriptomic and metabolomic**	**2 (1.6)**
Affymetrix transcriptomics arrays	
NMR	

### CKD Risk Categories

**FIGURE 2:**
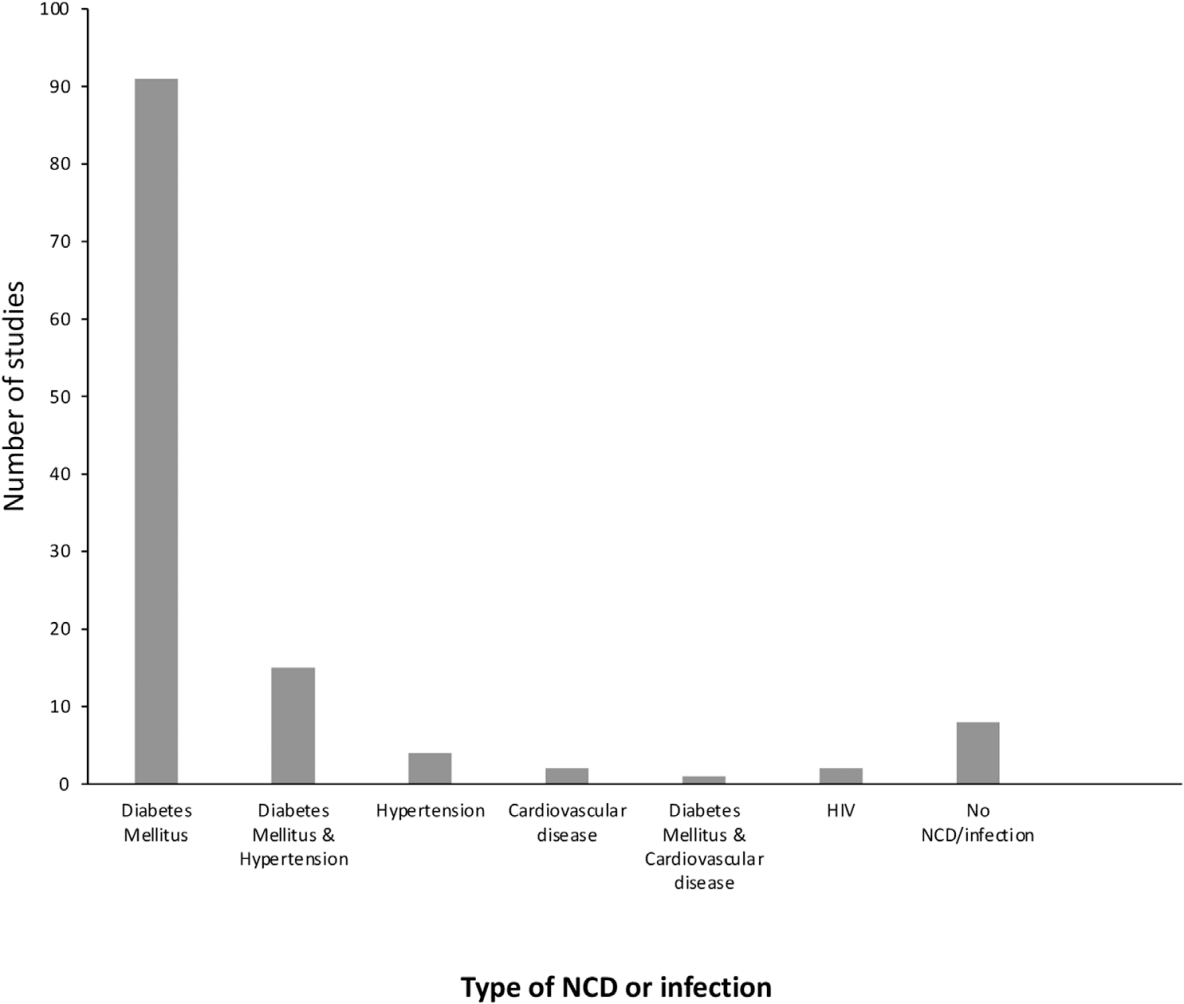
The number of papers describing each of the comorbidities associated with CKD.

The most common risk factors associated with CKD were diabetes mellitus (DM) (either type 1 diabetes, type 2 diabetes or both) (*n* = 91), followed by hypertension (*n* = 4), cardiovascular disease (*n* = 2), and infection (HIV) (*n* = 2). Sixteen studies reported on more than one risk factor and eight studies did not report on any risk factors (Figure 2).

### Biomarkers Associated With Risk, Early Detection, and Progression of CKD

Whilst it was not feasible to discuss each biomarker identified for each study across all ‘omics platforms, a list of selected studies that identified biomarkers/indicators involved in risk, early detection and prediction of progression, and those that explored potential disease pathways, are shown in Table 4.

**TABLE 4:** Studies that identified biomarkers/indicators for early detection of CKD, prediction of CKD progression, and disease pathways.

Author and year	Type of ‘omic platform	‘Omic technique	Sample size	Biomarker/indicator of disease	Effect estimate OR/*p* value	Clinical phenotype/outcome	What does the biomarker inform on?
Kao et al. (2008)	Genomic	1,354 SNP panel	2,178	rs16996674 (*MYH9*)	3.10	Non-diabetic ESKD	Susceptibility/risk
				rs16996677 (*MYH9*)	3.03		
				rs5756152 (*MYH9*)	2.59		
Shlush et al. (2010)	Genomic	2,016 SNP panel	833	rs7286127 (chromosome 22)	≤10^–3^	Non-diabetic ESKD	Susceptibility/risk
				rs5756133 (chromosome 22)	≤10^–3^		
Igo Jr et al. (2011)	Genomic	Illumina IV panel of 5,500 SNPs	3,972	rs5769116 (10 Mb distal to *MYH9/APOL1*)	1.5 × 10^–3^	DN	Susceptibility/risk
Morris et al. (2019)	Genomic	A variety of GWAS arrays	312,468	rs77924615 (*UMOD*)	1.5 × 10^–54^	CKD	Susceptibility/risk/disease pathway
Fan et al. (2020)	Transcriptomic	RNA-sequencing	37	*RDH8*	0.02	DN	Disease pathway
				*RDH12*	0.03		
				*RBP4*	0.03		
Øvrehus et al. (2015)	Proteomic	CE-MS	35	CKD273	AUC of 0.977^a^	DN and Hypertension associated kidney disease	Early detection
Siwy et al. (2014)	Proteomic	CE-MS	165	CKD273	AUC of 0.95–1.0^a^ <0.05	DN	Early detection
Lindhardt et al. (2017)	Proteomic	CE-MS	737	CKD273	AUC of 0.84^a^ < 1.0 × 10^–4^	DN	Early detection
Roscioni et al. (2013)	Proteomic	CE-MS	88	CKD273	1.35	DN	Progression
Tofte et al. (2020)	Proteomic	CE-MS	1,775	CKD273	<1.0 × 10^–4^	DN	Progression
Gonzalez-Calero et al. (2016)	Metabolomic	Nuclear magnetic resonance (NMR)	118	Pantothenate	<1.0 × 10^–4^	Hypertension associated kidney disease	Early detection
Ma et al. (2020)	Metabolomic	Liquid chromatography-mass spectrometry	68	Pantothenate	5.0 × 10^–3^	DN	Progression/disease pathway
Lecamwasam et al. (2020)	Epigenomic	Illumina Infinium MethylationEPIC BeadChip	119	cg17944885	0.01	DN	Progression

a Diagnostic accuracy measured as area under receiver operating characteristic curve.

#### Genetic Variants Associated With Risk for CKD

The genes implicated in risk for CKD were non–muscle myosin heavy chain 9 (*MYH9*), apolipoprotein L1 (*APOL1*) and uromodulin (*UMOD*)*.* Kao et al. (2008) performed a genome-wide admixture scan in 2,178 individuals with European and African ancestry. They identified three SNPs (rs16996674, rs16996677, and rs5756152) in the *MYH9* gene that were associated with a ∼3 times greater risk of nondiabetic ESKD in African Americans compared to European Americans. Similarly, Shlush et al. (2010) identified two SNPs (rs7286127 and rs5756133) associated with nondiabetic ESKD on chromosome 22 in the region that contains the *MYH9* gene, as well as the neighbouring *APOL1* gene. In addition, Igo et al. (2011) identified one SNP (rs5769116) 10 Mb distal to *MYH9/APOL1* that was associated with DN. Morris et al. (2019) performed an association study of eGFR, a measure of kidney function, in 312,468 individuals of diverse ancestry. They identified a highly significant SNP (rs77924615; *p* = 1.5 × 10^–54^) in the *UMOD* gene associated with eGFR.

#### Biomarkers Associated With Early Detection of CKD

The proteomic CKD273 biomarker panel and metabolite pantothenic acid (PA) could potentially play a role in the early detection of CKD. Three proteomic studies by Siwy et al*.* (2014), Øvrehus et al*.* (2015), and Lindhardt et al*.* (2017) assessed the ability of a proteomic CKD273 biomarker panel to predict DN with diagnostic accuracy, measured as area under receiver operating characteristic curve, ranging from 0.84 to 1.0. All three proteomic studies used the CE-MS approach with sample sizes ranging from 35 to 737. In addition, Gonzalez-Calero et al. (2016) identified the metabolite PA as a potential predictor of albuminuria development in 118 hypertensive patients.

#### Biomarkers Associated With Progression of CKD

The proteomic CKD273 biomarker panel, metabolite PA, and methylation probe cg17944885 were suggested to be involved in progression of CKD. Two proteomic studies, Roscioni et al. (2013) and Tofte et al. (2020) found that a high CKD273 risk score was able to predict progression of microalbuminuria with an odds ratio (OR) of 1.35 and *p* value of <1.0 × 10^–4^, respectively. In addition to the metabolite PA being a potential predictor of albuminuria development in hypertensive patients, it was also associated with DN progression (*p* = 5.0 × 10^–3^) (Ma et al., 2020). Lecamwasam et al. (2020) compared methylation profiles between early and late stages of DN. They found that the methylation probe cg17944885 was associated with progressive renal dysfunction (*p* = 0.01 after adjustment for multiple testing).

#### Biomarkers Exploring Potential Disease Pathways

Genes of the retinoic acid pathway, *UMOD* and the metabolite PA provided insight into potential pathways involved in CKD. *UMOD* was specifically expressed in epithelial cells of the ascending loop of Henle in the kidney. This points to the role of uromodulin in kidney physiology (Morris et al., 2019). Genes of the retinoic acid pathway are essential for retinoic acid (RA) synthesis and impairment of RA synthesis leads to progression of kidney disease (Li et al., 2014). Pathway analysis of PA revealed that altered PA synthesis and PA-related metabolism could lead to DN (Ma et al., 2020).

## Discussion

In this scoping review the first objective was to identify studies on potential biomarkers associated with CKD. The biomarkers that were most commonly associated with risk or early detection of CKD were genes and genetic variants on chromosomes 16 and 22 (Kao et al., 2008; Shlush et al., 2010; Igo et al., 2011; Liao et al., 2014; Taira et al., 2018; Morris et al., 2019), the CKD273 biomarker panel (consisting of proteins such as collagen fragments, uromodulin, α-1-antitrypsin, transthyretin and β-2-microglobulin) (Good et al., 2010; Siwy et al., 2014; Øvrehus et al., 2015; Lindhardt et al., 2017), and the metabolite PA (also known as pantothenate or vitamin B5) (Gonzalez-Calero et al., 2016). Majority of these potential biomarkers are exploratory, having been identified in small discovery studies and have not been validated, with the exception of the CKD273 biomarker panel.

It is well known from studies in the United States that individuals with African ancestry are at greater risk for developing incident CKD and progressing to ESKD, compared to other population groups (Perneger et al., 1995; Tarver-Carr et al., 2002; Saran et al., 2020). Much of this risk was attributed to genetic variants in the *MYH9/APOL1* gene region on chromosome 22 that were strongly associated with non-diabetic ESKD (Kao et al., 2008; Shlush et al., 2010). The *MYH9* and *APOL1* genes are tightly linked on chromosome 22, and fine mapping studies revealed that the risk variants were actually in the *APOL1* gene and not in the *MYH9* gene, as originally thought (Genovese et al., 2010; Tzur et al., 2010). Zhang et al. (2016) developed and validated an *APOL1* genotyping assay as a precision medicine genetic test for non-diabetic CKD risk prediction (Zhang et al., 2016). There are still ongoing clinical trials (http://www.clinicaltrials.gov; identifiers NCT02234063, NCT04191824 and NCT04910867), assessing the clinical utility of *APOL1* genetic testing. Implementation of an *APOL1* genotyping assay in a clinical setting could lead to a cost-effective screening program for African Americans who are at high risk for developing CKD and progressing to ESKD.

Studies have suggested the *UMOD* gene located on chromosome 16 as a candidate gene for CKD (Köttgen et al., 2009; Köttgen et al., 2010; Pattaro et al., 2016). Not only does it have a large effect on eGFR decline and CKD risk, the consistency of the effect is seen across different ethnicities (Devuyst et al., 2017). Morris et al. (2019) identified rs77924615 (*p* = 1.5 × 10^–54^), located in *UMOD*, as the main variant driving eGFR association. This variant was associated with decreased eGFR and increased *UMOD* expression (Morris et al., 2019).

Due to the multifactorial nature of CKD, a combination approach of several biomarkers is likely to be more predictive than a single biomarker. Compared to currently available markers such as reduced eGFR and albuminuria, the CKD273 biomarker panel enables more accurate diagnosis and earlier detection of CKD. For example, in one study, CKD273 enabled detection of CKD with 95% sensitivity, 100% specificity and an excellent diagnostic accuracy of 0.977 (95% confidence interval (CI) 0.930–1.000) (Øvrehus et al., 2015). In another study, CKD273 had the ability to detect DN in individuals with type 2 diabetes, with diagnostic accuracy ranging from 0.95 to 1.0 across nine study sites (Siwy et al., 2014). This same biomarker panel was able to predict the development of microalbuminuria in normoalbuminuric type 2 diabetic patients, with a diagnostic accuracy of 0.79 (95% CI 0.75–0.84; *p* < 1.0 × 10^–4^) (Lindhardt et al., 2017). The CKD273 biomarker panel includes clinically relevant proteins such as uromodulin and β-2-microglobulin, which are suggested to be markers of early renal damage (Torffvit and Agardh, 1993; Torffvit et al., 1999; Schlatzer et al., 2012; Zeng et al., 2014; Alaje Abiodun et al., 2016). These findings demonstrate the utility of CKD273 in early non-invasive renal risk assessment and may help to prevent unnecessary biopsies. While CKD273 has been validated for DN, its utility for hypertension-associated CKD is yet to be established. The CKD273 biomarker can assist clinicians in decision-making for more frequent check-ups in high-risk patients and guide therapeutic options to prevent long-term chronic conditions. Although the CKD273 biomarker panel has been supported by the FDA (https://www.fda.gov/files/drugs/published/Biomarker-Letter-of-Support_Mischak.pdf), its utility has yet to be established in African populations and the African diaspora. Furthermore, the current cost of screening using the CKD273 biomarker panel is €3,053 per patient making it unaffordable in resource-poor settings such as SSA (Critselis et al., 2018).

CKD273, PA, and methylation probe cg17944885 are suggested to be potential biomarkers associated with CKD progression. In addition to PA being used to predict albuminuria in hypertensive normoalbuminuric patients, it is suggested to be a promising biomarker for predicting diabetes-induced kidney impairment (Gonzalez-Calero et al., 2016; Ma et al., 2020). Methylation marker cg17944885 showed an increasing gradient of methylation with progressive renal dysfunction (Lecamwasam et al., 2020). A high CKD273 risk score was independently associated with faster progression of albuminuria in two separate studies (Roscioni et al., 2013; Tofte et al., 2020). The ability to identify individuals at high-risk for progression has important clinical implications. For those patients at a high-risk for progression, it is suggested that blood pressure be controlled to less than 130/80 mmHg. There are many therapeutic options to control blood pressure including angiotensin-converting enzyme (ACE) inhibitors, angiotensin-receptor blockers (ARBs) or a combination of ACE inhibitors or ARBs with a diuretic (Nguyen et al., 2010). Meanwhile, management of glucose and lipid levels are also suggested to delay the development of chronic complications in DN patients.

The second objective of the scoping review was to explore potential pathways involved in CKD. Using expression quantitative trait loci (eQTL) analysis, *UMOD* expression was mapped to epithelial cells lining the thick ascending limb of the loop of Henle (Morris et al., 2019). Studies on transgenic mice suggested that overexpression of uromodulin causes abnormal activation of the sodium-potassium-chloride transporter (NKCC2) involved in sodium chloride (NaCl) reabsorption by the thick ascending limb segment, leading to salt-sensitive hypertension and renal lesions (Trudu et al., 2013). Mice treated with a single dose of furosemide, a diuretic that targets NKCC2, exhibited a significant reduction in blood pressure (Trudu et al., 2013). This finding suggests that NKCC2 is a potential target for hypertension-induced CKD intervention.

Fan et al. (2020) compared gene expression profiles in three groups; controls, early DN and advanced DN (Fan et al., 2020). They identified genes of the retinoic acid pathway including retinol dehydrogenase 8 (*RDH8*)*,* retinol dehydrogenase 12 (*RDH12*), and retinol binding protein 4 (*RBP4*) with increased expression in early DN but decreased expression in advanced DN. These findings suggest that RA might have kidney protective effects in the early stage of DN that slow the progression to advanced DN. A previous study suggested that downregulation of enzymes retinol dehydrogenase 9 (RDH9) and retinol dehydrogenase 1 (RDH1) essential for RA synthesis causes impaired local synthesis of RA in the kidney that contributes to the progression of kidney disease (Li et al., 2014). These results point to restoration of retinoic acid synthesis as a potential therapy to slow the progression of kidney disease. There is an ongoing clinical trial assessing the efficacy of RA isotretinoin for treatment of various podocyte diseases including collapsing glomerulopathy (http://www.clinicaltrials.gov; identifier NCT00098020).

The metabolite PA is suggested to be a predictive biomarker for development of CKD and progression in hypertensive and diabetic patients (Gonzalez-Calero et al., 2016; Ma et al., 2020). PA (also known as vitamin B5) is an essential vitamin and dietary factor, and has long been recognised as an essential cofactor of biochemical reactions in numerous physiological processes (Patassini et al., 2019). Pathway analysis results showed PA synthesis and PA-related metabolism were disturbed in individuals with DN (Ma et al., 2020). Since mammalian cells are unable to synthesize vitamin B5, they require it to be generated by the intestinal microbiome or to be part of the diet, resulting in a potential therapeutic target for DN treatment (Daugherty et al., 2002). A previous study in a type 2 diabetes rat model has shown that a vitamin B5 supplement restored body weight and blood pressure whilst also decreasing blood glucose levels (Demirci et al., 2014). In addition, this study also reported that a vitamin B5 supplement may reduce diabetes-related oxidative stress and prevent endothelial cell injury (Demirci et al., 2014). However, further studies are required to determine the efficacy of a vitamin B5 supplement in reducing blood pressure and glucose levels in humans.

The translation of ‘omics research into disease-related biomarkers for use in clinical practice is a complicated, time-consuming, and expensive process. Some challenges include clinical heterogeneity of individuals with a particular diagnosis, different standards of evidence to evaluate the validity of the biomarker, and a lengthy developmental pathway from biomarker discovery to clinical utility (Omenn et al., 2012). The diagnostic and therapeutic potential of the biomarker has to be carefully assessed before being considered for introduction into the clinic, and additional uses should be considered. For example, the CKD273 biomarker panel is available as a commercial *in vitro* diagnostic test and is a good example of a “bench-to-bedside” approach (Pontillo and Mischak, 2017). In addition to its diagnostic and prognostic value, CKD273 also has the potential to assess the impact (outcome) of therapeutic interventions (Haubitz et al., 2009). For example, in one study, CKD273 risk scores were significantly reduced (from 0.721 at visit 2 to 0.277 at visit 9), below the previously defined threshold (0.343) after 2 years of treatment with irbesartan (an antihypertensive and antiproteinuric drug) (Andersen et al., 2010).

Low-cost genomics-informed approaches to disease risk prediction pose great promise for low- and middle-income countries, where the morbidity and mortality of non-communicable diseases such as CKD are disproportionately high. However, there are many factors that hinder the implementation of a genomic medicine healthcare model for CKD in resource limited settings. These include population-specific research to validate predictive accuracy and clinical utility, a low critical mass of skilled scientists, insufficiently equipped diagnostic research laboratories and a lagging public health policy framework (Tekola-Ayele and Rotimi, 2015). For example, the paucity of genomics research in individuals from Africa has limited knowledge of clinically relevant CKD genetic variants in these populations. The clinical implementation of genomic medicine requires more than just developing a diagnostic or screening test. To have a functional genomic medicine healthcare model for CKD, costly technology and skill-strengthening among scientists in the fields of medical genetics, genetic counselling, genetic epidemiology, bioinformatics and computational biology are required. In addition, legal and ethical frameworks also need to be strengthened in local contexts (Tekola-Ayele and Rotimi, 2015; Pereira et al., 2021).

The strengths of the review include the novelty of the topic and the assessment of the literature on candidate biomarkers for early detection of CKD and prediction of progression. Several potential biomarkers are highlighted and may be useful in developing screening programs for CKD, however, they need validation in different clinical settings and ethnic backgrounds. In addition, this review has shown that ‘omics technologies can be used to explore potential disease mechanisms and therapeutic targets for treatment. There were some limitations in this scoping review. Despite internationally accepted guidelines, studies used various metrics to define CKD and potential sex differences in the manifestation of CKD were not examined. The sample sizes were usually small, and results may not be transferable to populations that were not included in the studies, for example African populations. Furthermore, it was difficult to compare the outcomes across studies because each study design was unique.

A few knowledge gaps were identified. While DM is a major risk factor for CKD in some regions of the world, reflected in the number of DN studies reported in the ‘omics literature, other regions such as Asia and Africa have other dominant risk factors such as hypertension and other contributing factors that remain to be identified. The major underlying causes of kidney disease in Africa are poorly understood and often attributed to hypertension which is likely to be secondary hypertension as a consequence of numerous insults. In this regard, there are relatively few ‘omics studies that focus on hypertension in the context of CKD in African populations (Alicic et al., 2017). There was a single study with individuals from West Africa, highlighting the lack of ‘omics research in continental Africans (Chen et al., 2007), despite many studies documenting the well-established genetic link to CKD in African Americans (*APOL1-*associated nephropathy) (George et al., 2018).

In sub-Saharan Africa, there are limited resources for treating individuals with CKD who progress to ESKD, rendering this condition fatal for those who are not treated. At least half of the patients who require ESKD treatment do not have access to treatment, and only 1.5% are predicted to receive renal replacement therapy (Ashuntantang et al., 2017). Approximately 75% of patients who begin dialysis will subsequently demise due to poor quality of dialysis or termination of dialysis based on its cost (Ashuntantang et al., 2017). This makes screening, early detection and the implementation of preventative interventions for CKD particularly relevant. Clinically relevant biomarkers will be useful to develop screening programs and potential therapeutic approaches to treat CKD in sub-Saharan Africa. Ideally the goal is to identify individuals at risk for developing CKD in the future and develop preventative intervention strategies that would delay onset and slow progression, by treating the underlying etiologies.

The reported ‘omics studies identified potential biomarkers associated with CKD. These biomarkers provide insight into the pathways involved in CKD and could possibly be used to guide diagnostic and treatment options. The biomarkers that have emerged through synthesis of existing literature provide evidence for effective approaches for early detection of CKD and prediction of progression in European populations. Their transferability to Africans and other ethnicities needs to be verified through research in relevant populations and assessment of diagnostic implementation in low-resourced healthcare settings. There is extensive regional and ethnic diversity in Africa, and the major causes of CKD appear to differ substantively from those in high-income settings, requiring population representative studies that address the local context and causes of CKD. In addition, many factors need to be addressed to facilitate effective genomic medicine healthcare models for CKD.

## Data Availability

The search terms and methodology are fully described in the paper and the list of papers included in the review are shown in the Supplementary Material.
