# Supplementary material for: The Use of ‘Omics for Diagnosing and Predicting Progression of Chronic Kidney Disease: A Scoping Review
Source: Front Genet. 2021 Nov 8;12:682929. doi: 10.3389/fgene.2021.682929 (PMC8606569; doi:10.3389/fgene.2021.682929)
Supplement: Supplementary file 1 [file DataSheet1.docx]

Supplementary Appendix A

List of 123 full-text papers assessed and included in the review.

1 Gil, R. B. *et al.* Increased urinary osmolyte excretion indicates chronic kidney disease severity and progression rate. *Nephrology Dialysis Transplantation* **33**, 2156-2164 (2018).

2 Hallan, S. *et al.* Metabolomics and gene expression analysis reveal down-regulation of the citric acid (TCA) cycle in non-diabetic CKD patients. *EBioMedicine* **26**, 68-77 (2017).

3 Fan, Y. *et al.* Erratum. Comparison of Kidney Transcriptomic Profiles of Early and Advanced Diabetic Nephropathy Reveals Potential New Mechanisms for Disease Progression. Diabetes 2019; 68: 2301–2314. *Diabetes* **69**, 797-797 (2020).

4 Perez-Hernandez, J. *et al.* Urinary-and Plasma-Derived Exosomes Reveal a Distinct MicroRNA Signature Associated With Albuminuria in Hypertension. *Hypertension* **77**, 960-971 (2021).

5 Monteiro, M. B. *et al.* Urinary Sediment Transcriptomic and Longitudinal Data to Investigate Renal Function Decline in Type 1 Diabetes. *Frontiers in Endocrinology* **11**, 238 (2020).

6 Roux, M. *et al.* Plasma levels of hsa-miR-152-3p are associated with diabetic nephropathy in patients with type 2 diabetes. *Nephrology Dialysis Transplantation* **33**, 2201-2207 (2018).

7 Nair, V. *et al.* A molecular morphometric approach to diabetic kidney disease can link structure to function and outcome. *Kidney international* **93**, 439-449 (2018).

8 Li, H. *et al.* Epigenetic regulation of RCAN1 expression in kidney disease and its role in podocyte injury. *Kidney international* **94**, 1160-1176 (2018).

9 Liu, J.-J. *et al.* Profiling of plasma metabolites suggests altered mitochondrial fuel usage and remodeling of sphingolipid metabolism in individuals with type 2 diabetes and kidney disease. *Kidney international reports* **2**, 470-480 (2017).

10 Gordin, D. *et al.* Characterization of glycolytic enzymes and pyruvate kinase M2 in type 1 and 2 diabetic nephropathy. *Diabetes Care* **42**, 1263-1273 (2019).

11 Kammer, M. *et al.* Integrative analysis of prognostic biomarkers derived from multiomics panels helps discrimination of chronic kidney disease trajectories in people with type 2 diabetes. *Kidney International* **96**, 1381-1388 (2019).

12 Smith, A. *et al.* Detecting Proteomic Indicators to Distinguish Diabetic Nephropathy from Hypertensive Nephrosclerosis by Integrating Matrix-Assisted Laser Desorption/Ionization Mass Spectrometry Imaging with High-Mass Accuracy Mass Spectrometry. *Kidney and Blood Pressure Research* **45**, 233-248 (2020).

13 Baldan-Martin, M. *et al.* Plasma Molecular Signatures in Hypertensive Patients With Renin–Angiotensin System Suppression: New Predictors of Renal Damage and De Novo Albuminuria Indicators. *Hypertension* **68**, 157-166 (2016).

14 Edfors, R. *et al.* Use of proteomics to identify biomarkers associated with chronic kidney disease and long‐term outcomes in patients with myocardial infarction. *Journal of Internal Medicine* **288**, 581-592 (2020).

15 Øvrehus, M. A., Zürbig, P., Vikse, B. E. & Hallan, S. I. Urinary proteomics in chronic kidney disease: diagnosis and risk of progression beyond albuminuria. *Clinical proteomics* **12**, 1-9 (2015).

16 Glazyrin, Y. E. *et al.* Proteomics-based machine learning approach as an alternative to conventional biomarkers for differential diagnosis of chronic kidney diseases. *International Journal of Molecular Sciences* **21**, 4802 (2020).

17 Dihazi, H. *et al.* Characterization of diabetic nephropathy by urinary proteomic analysis: identification of a processed ubiquitin form as a differentially excreted protein in diabetic nephropathy patients. *Clinical chemistry* **53**, 1636-1645 (2007).

18 RAJESH, K., DHEEBA, B., SAMPATHKUMAR, P. & SIVAKUMAR, R. PROTEOMIC ANALYSIS OF HUMAN BLOOD AND URINE IN DIABETIC NEPHROPATHY.

19 Carlsson, A. C. *et al.* Use of a proximity extension assay proteomics chip to discover new biomarkers associated with albuminuria. *European Journal of Preventive Cardiology* **24**, 340-348 (2017).

20 Verbeke, F. *et al.* The urinary proteomics classifier chronic kidney disease 273 predicts cardiovascular outcome in patients with chronic kidney disease. *Nephrology Dialysis Transplantation* **36**, 811-818 (2021).

21 Subasi, E. *et al.* A classification model to predict the rate of decline of kidney function. *Frontiers in Medicine* **4**, 97 (2017).

22 Ngo, D. *et al.* Circulating testican-2 is a podocyte-derived marker of kidney health. *Proceedings of the National Academy of Sciences* **117**, 25026-25035 (2020).

23 Gu, Y.-M. *et al.* The urinary proteome as correlate and predictor of renal function in a population study. *Nephrology Dialysis Transplantation* **29**, 2260-2268 (2014).

24 So, E. J., Kim, H. J. & Kim, C. W. Proteomic analysis of human proximal tubular cells exposed to high glucose concentrations. *PROTEOMICS–Clinical Applications* **2**, 1118-1126 (2008).

25 Pontillo, C. *et al.* Prediction of chronic kidney disease stage 3 by CKD273, a urinary proteomic biomarker. *Kidney international reports* **2**, 1066-1075 (2017).

26 Caseiro, A. *et al.* Pursuing type 1 diabetes mellitus and related complications through urinary proteomics. *Translational Research* **163**, 188-199 (2014).

27 Merchant, M. L. *et al.* Plasma kininogen and kininogen fragments are biomarkers of progressive renal decline in type 1 diabetes. *Kidney international* **83**, 1177-1184 (2013).

28 Millioni, R. *et al.* Abnormal cytoskeletal protein expression in cultured skin fibroblasts from type 1 diabetes mellitus patients with nephropathy: A proteomic approach. *PROTEOMICS–Clinical Applications* **2**, 492-503 (2008).

29 Overgaard, A. J. *et al.* Quantitative iTRAQ-based proteomic identification of candidate biomarkers for diabetic nephropathy in plasma of type 1 diabetic patients. *Clinical proteomics* **6**, 105-114 (2010).

30 Overgaard, A. J. *et al.* Plasma proteome analysis of patients with type 1 diabetes with diabetic nephropathy. *Proteome science* **8**, 4 (2010).

31 Schlatzer, D. *et al.* Novel urinary protein biomarkers predicting the development of microalbuminuria and renal function decline in type 1 diabetes. *Diabetes care* **35**, 549-555 (2012).

32 Vitova, L. *et al.* Early urinary biomarkers of diabetic nephropathy in type 1 diabetes mellitus show involvement of kallikrein-kinin system. *BMC nephrology* **18**, 1-10 (2017).

33 Gianazza, E. *et al.* Different expression of fibrinopeptide A and related fragments in serum of type 1 diabetic patients with nephropathy. *Journal of proteomics* **73**, 593-601 (2010).

34 Wu, J., Chen, Y.-D., Yu, J.-K., Shi, X.-L. & Gu, W. Analysis of urinary proteomic patterns for type 2 diabetic nephropathy by ProteinChip. *diabetes research and clinical practice* **91**, 213-219 (2011).

35 Zhang, S., Li, X., Luo, H., Fang, Z.-Z. & Ai, H. Role of aromatic amino acids in pathogeneses of diabetic nephropathy in Chinese patients with type 2 diabetes. *Journal of Diabetes and its Complications* **34**, 107667 (2020).

36 Lu, H., Deng, S., Zheng, M. & Hu, K. iTRAQ plasma proteomics analysis for candidate biomarkers of type 2 incipient diabetic nephropathy. *Clinical proteomics* **16**, 1-11 (2019).

37 Lindhardt, M. *et al.* Urinary proteomics predict onset of microalbuminuria in normoalbuminuric type 2 diabetic patients, a sub-study of the DIRECT-Protect 2 study. *Nephrology Dialysis Transplantation* **32**, 1866-1873 (2017).

38 Guillén-Gómez, E. *et al.* Urinary proteome analysis identified neprilysin and VCAM as proteins involved in diabetic nephropathy. *Journal of diabetes research* **2018** (2018).

39 Jiang, H. *et al.* Identification of urinary soluble E‐cadherin as a novel biomarker for diabetic nephropathy. *Diabetes/metabolism research and reviews* **25**, 232-241 (2009).

40 Lim, S. *et al.* Adipocytokine zinc α2 glycoprotein (ZAG) as a novel urinary biomarker for normo‐albuminuric diabetic nephropathy. *Diabetic Medicine* **29**, 945-949 (2012).

41 Yang, J.-K. *et al.* Urine proteome specific for eye damage can predict kidney damage in patients with type 2 diabetes: a case-control and a 5.3-year prospective cohort study. *Diabetes care* **40**, 253-260 (2017).

42 Yang, Y. *et al.* Predicting diabetic nephropathy by serum proteomic profiling in patients with type 2 diabetes. *Wiener klinische Wochenschrift* **127**, 669-674 (2015).

43 Alkhalaf, A. *et al.* Multicentric validation of proteomic biomarkers in urine specific for diabetic nephropathy. *PloS one* **5**, e13421 (2010).

44 Roscioni, S. *et al.* A urinary peptide biomarker set predicts worsening of albuminuria in type 2 diabetes mellitus. *Diabetologia* **56**, 259-267 (2013).

45 Ahn, J. M., Kim, B. G., Yu, M. H., Lee, I. K. & Cho, J. Y. Identification of diabetic nephropathy‐selective proteins in human plasma by multi‐lectin affinity chromatography and LC‐MS/MS. *PROTEOMICS–Clinical Applications* **4**, 644-653 (2010).

46 Rao, P. V. *et al.* Proteomic identification of urinary biomarkers of diabetic nephropathy. *Diabetes care* **30**, 629-637 (2007).

47 Marikanty, R. *et al.* Identification of urinary proteins potentially associated with diabetic kidney disease. *Indian journal of nephrology* **26**, 434 (2016).

48 Yeh, S.-H. *et al.* Differentiation of type 2 diabetes mellitus with different complications by proteomic analysis of plasma low abundance proteins. *Journal of Diabetes & Metabolic Disorders* **15**, 1-7 (2015).

49 Tofte, N. *et al.* Characteristics of high‐and low‐risk individuals in the PRIORITY study: urinary proteomics and mineralocorticoid receptor antagonism for prevention of diabetic nephropathy in Type 2 diabetes. *Diabetic Medicine* **35**, 1375-1382 (2018).

50 Currie, G. E. *et al.* Urinary proteomics for prediction of mortality in patients with type 2 diabetes and microalbuminuria. *Cardiovascular diabetology* **17**, 50 (2018).

51 Bellei, E. *et al.* Proteomic analysis of early urinary biomarkers of renal changes in type 2 diabetic patients. *PROTEOMICS–Clinical Applications* **2**, 478-491 (2008).

52 Kim, M.-R., Yu, S.-A., Kim, M.-Y., Choi, K. M. & Kim, C.-W. Analysis of glycated serum proteins in type 2 diabetes patients with nephropathy. *Biotechnology and bioprocess engineering* **19**, 83-92 (2014).

53 Petrica, L. *et al.* Glycated peptides are associated with proximal tubule dysfunction in type 2 diabetes mellitus. *International journal of clinical and experimental medicine* **8**, 2516 (2015).

54 Siwy, J. *et al.* Multicentre prospective validation of a urinary peptidome-based classifier for the diagnosis of type 2 diabetic nephropathy. *Nephrology Dialysis Transplantation* **29**, 1563-1570 (2014).

55 Hung, P.-H. *et al.* Proteomic identification of plasma biomarkers in type 2 diabetic nephropathy. *Journal of Integrated OMICS* **1**, 151-156 (2010).

56 Bellei, E. *et al.* Urinary proteomics in biomarker discovery of kidney-related disorders: diabetic nephropathy and drug-induced nephrotoxicity in chronic headache. *Ejifcc* **29**, 290 (2018).

57 Kaburagi, Y. *et al.* Urinary afamin levels are associated with the progression of diabetic nephropathy. *Diabetes research and clinical practice* **147**, 37-46 (2019).

58 Papale, M. *et al.* Urine proteome analysis may allow noninvasive differential diagnosis of diabetic nephropathy. *Diabetes care* **33**, 2409-2415 (2010).

59 Patel, D. N. & Kalia, K. Characterization of low molecular weight urinary proteins at varying time intervals in type 2 diabetes mellitus and diabetic nephropathy patients. *Diabetology & metabolic syndrome* **11**, 39 (2019).

60 Otu, H. H. *et al.* Prediction of diabetic nephropathy using urine proteomic profiling 10 years prior to development of nephropathy. *Diabetes care* **30**, 638-643 (2007).

61 Lindhardt, M. *et al.* Predicting albuminuria response to spironolactone treatment with urinary proteomics in patients with type 2 diabetes and hypertension. *Nephrology Dialysis Transplantation* **33**, 296-303 (2018).

62 Pena, M. J. *et al.* Plasma proteomics classifiers improve risk prediction for renal disease in patients with hypertension or type 2 diabetes. *Journal of hypertension* **33**, 2123-2132 (2015).

63 Lewandowicz, A. *et al.* Changes in urine proteome accompanying diabetic nephropathy progression. *Polskie Archiwum Medycyny Wewnętrznej* **125**, 27-38 (2015).

64 Tofte, N. *et al.* Early detection of diabetic kidney disease by urinary proteomics and subsequent intervention with spironolactone to delay progression (PRIORITY): a prospective observational study and embedded randomised placebo-controlled trial. *The Lancet Diabetes & Endocrinology* **8**, 301-312 (2020).

65 Li, Y. *et al.* Genome-wide association studies of metabolites in patients with CKD identify multiple loci and illuminate tubular transport mechanisms. *Journal of the American Society of Nephrology* **29**, 1513-1524 (2018).

66 Luo, S. *et al.* Genome-wide association study of serum metabolites in the African American Study of Kidney Disease and Hypertension. *Kidney international* (2021).

67 Hu, J.-R. *et al.* Serum metabolites are associated with all-cause mortality in chronic kidney disease. *Kidney international* **94**, 381-389 (2018).

68 Luo, S. *et al.* Serum metabolomic alterations associated with proteinuria in CKD. *Clinical Journal of the American Society of Nephrology* **14**, 342-353 (2019).

69 Kwan, B. *et al.* Metabolomic markers of kidney function decline in patients with diabetes: Evidence From the Chronic Renal Insufficiency Cohort (CRIC) study. *American Journal of Kidney Diseases* **76**, 511-520 (2020).

70 Huang, J. *et al.* Machine Learning Approaches Reveal Metabolic Signatures of Incident Chronic Kidney Disease in Individuals with Prediabetes and Type 2 Diabetes. *Diabetes* **69**, 2756-2765 (2020).

71 Yu, B. *et al.* Serum metabolomic profiling and incident CKD among African Americans. *Clinical Journal of the American Society of Nephrology* **9**, 1410-1417 (2014).

72 Duranton, F. *et al.* Plasma and urinary amino acid metabolomic profiling in patients with different levels of kidney function. *Clinical Journal of the American Society of Nephrology* **9**, 37-45 (2014).

73 Feng, Q., Li, Y., Yang, Y. & Feng, J. Urine metabolomics analysis in patients with normoalbuminuric diabetic kidney disease. *Frontiers in Physiology* **11** (2020).

74 Kimura, T. *et al.* Identification of biomarkers for development of end-stage kidney disease in chronic kidney disease by metabolomic profiling. *Scientific reports* **6**, 1-8 (2016).

75 Lee, J. *et al.* Changes in serum metabolites with the stage of chronic kidney disease: Comparison of diabetes and non-diabetes. *Clinica Chimica Acta* **459**, 123-131 (2016).

76 Toyohara, T. *et al.* Metabolomic profiling of uremic solutes in CKD patients. *Hypertension research* **33**, 944-952 (2010).

77 Gonzalez-Calero, L. *et al.* Hypertensive patients exhibit an altered metabolism. A specific metabolite signature in urine is able to predict albuminuria progression. *Translational Research* **178**, 25-37. e27 (2016).

78 Rhee, E. P. *et al.* Metabolomics of chronic kidney disease progression: A case-control analysis in the chronic renal insufficiency cohort study. *American journal of nephrology* **43**, 366-374 (2016).

79 Masania, J. *et al.* Urinary Metabolomic Markers of Protein Glycation, Oxidation, and Nitration in Early-Stage Decline in Metabolic, Vascular, and Renal Health. *Oxidative medicine and cellular longevity* **2019** (2019).

80 Manca, M. L. *et al.* Differential metabolomic signatures of declining renal function in types 1 and 2 diabetes. *Nephrology Dialysis Transplantation* (2020).

81 Sharma, K. *et al.* Metabolomics reveals signature of mitochondrial dysfunction in diabetic kidney disease. *Journal of the American Society of Nephrology* **24**, 1901-1912 (2013).

82 Moon, S. *et al.* Circulating short and medium chain fatty acids are associated with normoalbuminuria in type 1 diabetes of long duration. *Scientific reports* **11**, 1-13 (2021).

83 Haukka, J. K. *et al.* Metabolomic profile predicts development of microalbuminuria in individuals with type 1 diabetes. *Scientific reports* **8**, 1-10 (2018).

84 Niewczas, M. A. *et al.* Circulating modified metabolites and a risk of ESRD in patients with type 1 diabetes and chronic kidney disease. *Diabetes Care* **40**, 383-390 (2017).

85 Tofte, N. *et al.* Metabolomic assessment reveals alteration in polyols and branched chain amino acids associated with present and future renal impairment in a discovery cohort of 637 persons with type 1 diabetes. *Frontiers in endocrinology* **10**, 818 (2019).

86 Ma, T. *et al.* UPLC-MS-based urine nontargeted metabolic profiling identifies dysregulation of pantothenate and CoA biosynthesis pathway in diabetic kidney disease. *Life Sciences* **258**, 118160 (2020).

87 Niewczas, M. A. *et al.* Uremic solutes and risk of end-stage renal disease in type 2 diabetes: metabolomic study. *Kidney international* **85**, 1214-1224 (2014).

88 Ng, D. *et al.* A metabolomic study of low estimated GFR in non-proteinuric type 2 diabetes mellitus. *Diabetologia* **55**, 499-508 (2012).

89 Tao, S. *et al.* Analysis of serum metabolomics among biopsy-proven diabetic nephropathy, type 2 diabetes mellitus and healthy controls. *RSC advances* **9**, 18713-18719 (2019).

90 Lin, H.-T. *et al.* 1H nuclear magnetic resonance (NMR)-based cerebrospinal fluid and plasma metabolomic analysis in type 2 diabetic patients and risk prediction for diabetic microangiopathy. *Journal of clinical medicine* **8**, 874 (2019).

91 Ibarra-González, I. *et al.* Optimization of kidney dysfunction prediction in diabetic kidney disease using targeted metabolomics. *Acta diabetologica* **55**, 1151-1161 (2018).

92 Chen, C.-J., Liao, W.-L., Chang, C.-T., Lin, Y.-N. & Tsai, F.-J. Identification of urinary metabolite biomarkers of type 2 diabetes nephropathy using an untargeted metabolomic approach. *Journal of proteome research* **17**, 3997-4007 (2018).

93 Solini, A. *et al.* Prediction of declining renal function and albuminuria in patients with type 2 diabetes by metabolomics. *The Journal of Clinical Endocrinology & Metabolism* **101**, 696-704 (2016).

94 Devi, S. *et al.* Acyl ethanolamides in Diabetes and Diabetic Nephropathy: Novel targets from untargeted plasma metabolomic profiles of South Asian Indian men. *Scientific reports* **9**, 1-11 (2019).

95 Rhee, E. P. *et al.* A combined epidemiologic and metabolomic approach improves CKD prediction. *Journal of the American Society of Nephrology* **24**, 1330-1338 (2013).

96 Sheng, X. *et al.* Systematic integrated analysis of genetic and epigenetic variation in diabetic kidney disease. *Proceedings of the National Academy of Sciences* **117**, 29013-29024 (2020).

97 Zawada, A. M. *et al.* SuperTAG methylation-specific digital karyotyping reveals uremia-induced epigenetic dysregulation of atherosclerosis-related genes. *Circulation: Cardiovascular Genetics* **5**, 611-620 (2012).

98 Smyth, L. J., Patterson, C. C., Swan, E. J., Maxwell, A. P. & McKnight, A. J. DNA methylation associated with diabetic kidney disease in Blood-Derived DNA. *Frontiers in cell and developmental biology* **8** (2020).

99 Qiu, C. *et al.* Cytosine methylation predicts renal function decline in American Indians. *Kidney international* **93**, 1417-1431 (2018).

100 Chen, J. *et al.* Epigenetic associations with estimated glomerular filtration rate among men with human immunodeficiency virus infection. *Clinical Infectious Diseases* **70**, 667-673 (2020).

101 Lecamwasam, A. *et al.* DNA methylation profiling identifies epigenetic differences between early versus late stages of diabetic chronic kidney disease. *Nephrology Dialysis Transplantation* (2020).

102 Smyth, L. J. *et al.* Assessment of differentially methylated loci in individuals with end-stage kidney disease attributed to diabetic kidney disease: an exploratory study. *Clinical epigenetics* **13**, 1-19 (2021).

103 Bell, C. G. *et al.* Genome-wide DNA methylation analysis for diabetic nephropathy in type 1 diabetes mellitus. *BMC medical genomics* **3**, 1-11 (2010).

104 Maghbooli, Z. *et al.* Aberrant DNA methylation patterns in diabetic nephropathy. *Journal of Diabetes & Metabolic Disorders* **13**, 1-8 (2014).

105 Zhang, H. *et al.* Correlation of CTGF gene promoter methylation with CTGF expression in type 2 diabetes mellitus with or without nephropathy. *Molecular medicine reports* **9**, 2138-2144 (2014).

106 VanderJagt, T. A., Neugebauer, M. H., Morgan, M., Bowden, D. W. & Shah, V. O. Epigenetic profiles of pre-diabetes transitioning to type 2 diabetes and nephropathy. *World journal of diabetes* **6**, 1113 (2015).

107 Shlush, L. I. *et al.* Admixture mapping of end stage kidney disease genetic susceptibility using estimated mutual information ancestry informative markers. *BMC medical genomics* **3**, 1-12 (2010).

108 Iyengar, S. K. *et al.* Genome-wide scans for diabetic nephropathy and albuminuria in multiethnic populations: the family investigation of nephropathy and diabetes (FIND). *Diabetes* **56**, 1577-1585 (2007).

109 Thomson, R. J. *et al.* New genetic loci associated with chronic kidney disease in an indigenous Australian population. *Frontiers in genetics* **10**, 330 (2019).

110 Hishida, A. *et al.* Genome-wide association study of renal function traits: results from the Japan Multi-Institutional Collaborative Cohort Study. *American journal of nephrology* **47**, 304-316 (2018).

111 Lin, B. M. *et al.* Whole genome sequence analyses of eGFR in 23,732 people representing multiple ancestries in the NHLBI trans-omics for precision medicine (TOPMed) consortium. *EBioMedicine* **63**, 103157 (2021).

112 Van Zuydam, N. R. *et al.* A genome-wide association study of diabetic kidney disease in subjects with type 2 diabetes. *Diabetes* **67**, 1414-1427 (2018).

113 Haukka, J. *et al.* Novel linkage peaks discovered for diabetic nephropathy in individuals with type 1 diabetes. *Diabetes* **70**, 986-995 (2021).

114 Palmer, N. D. *et al.* A genome-wide association search for type 2 diabetes genes in African Americans. *PloS one* **7**, e29202 (2012).

115 Huang, Y. *et al.* SNPs in PRKCA‐HIF1A‐GLUT1 are associated with diabetic kidney disease in a Chinese Han population with type 2 diabetes. *European journal of clinical investigation* **50**, e13264 (2020).

116 Liao, L.-N. *et al.* Identified single-nucleotide polymorphisms and haplotypes at 16q22. 1 increase diabetic nephropathy risk in Han Chinese population. *BMC genetics* **15**, 1-11 (2014).

117 Taira, M. *et al.* A variant within the FTO confers susceptibility to diabetic nephropathy in Japanese patients with type 2 diabetes. *PloS one* **13**, e0208654 (2018).

118 Kao, W. L. *et al.* MYH9 is associated with nondiabetic end-stage renal disease in African Americans. *Nature genetics* **40**, 1185-1192 (2008).

119 Igo Jr, R. P. *et al.* Genomewide linkage scan for diabetic renal failure and albuminuria: the FIND study. *American journal of nephrology* **33**, 381-389 (2011).

120 Morris, A. P. *et al.* Trans-ethnic kidney function association study reveals putative causal genes and effects on kidney-specific disease aetiologies. *Nature communications* **10**, 1-14 (2019).

121 Pezzolesi, M. G. *et al.* Genome-wide association scan for diabetic nephropathy susceptibility genes in type 1 diabetes. *Diabetes* **58**, 1403-1410 (2009).

122 Rogus, J. J. *et al.* High-density single nucleotide polymorphism genome-wide linkage scan for susceptibility genes for diabetic nephropathy in type 1 diabetes: discordant sibpair approach. *Diabetes* **57**, 2519-2526 (2008).

123 Chen, G. *et al.* A genome-wide search for linkage to renal function phenotypes in West Africans with type 2 diabetes. *American journal of kidney diseases* **49**, 394-400 (2007).
